# Scarification in Nasal Hypertrophy

**Published:** 1887-08

**Authors:** A. G. Hobbs

**Affiliations:** Atlanta, Georgia


### SCARIFICATION IN NASAL HYPERTROPHY.

By A. G. Hobbs, M. D., Atlanta, Georgia.

**[Read at St. Louis, Mo., before the American Rhinological Association.]**

During the last few years I have resorted to this means of reducing so-called mucous membrane hypertrophies found over the turbinated bones, which upon the suggestion of Dr. Rumbold are now collectively called *turbinated processes*. I have used it only as an auxillary to the ordinary spray treatment of vaseline, containing pure terebene, eucalyptol, sandalwood oil or pinus canadensis in proper proportions, in hypertrophied nasal catarrh; but I am confident that in a number of cases, it has not only hastened the cure but has enabled me to succeed where other means would have failed.

When the swelling is extensive enough to be snared, I have not relied wholly upon the scarifications but have only resorted to that means to reduce any small protrusions that may be left after the use of the snare.

After the part to be punctured is well cocanized by holding a cotton probe that has been saturated in a cocaine solution against it for two or three minutes, a sharp pointed narrow bistoury is introduced at the base of the process parallel with the nasal cartilage and pushed through its appex. The bleeding is usually profuse, varying from a drachm to an ounce. I always prefer to see it profuse. When the blood has ceased to flow the nose should be well sprayed with medicated vaseline. The second or third day after the cutting a depression will be seen in the line of the cicatrix which is somewhat lighter in color than the surrounding tissues. Then if a smaller puncture be made on one or both sides of the depression, the nasal stenosis will be much relieved. The operation should be attended with little or no pain if the part is properly cocanized. I have found that this means is not applicable to all turbinated enlargements, for example: if the hypertrophy is a true one and is consequently hard and resisting to the probe, but little blood will issue from the puncture and the result will be unsatisfactory.

The cases that I find best adapted to this treatment, are those in which the turbinated processes are œdematous in character and inclined to point pressing against or barely touching the protrusions upon the opposite side. If the two apices which may be touching are punctured to-day, to-morrow or the next day, they will be 1-16 to 1-8 of an inch apart.

The resultant eschar is so narrow, having been produced by an incised wound, that it cannot interfere with the functions of the mucous membrane to any appreciab e extent.

It would seem that these protrusions are hyperplastic and not hypertrophic in character for the reason that they are softer, contain more blood, and are more easily pressed down by the cicatrix than a true hypertrophy of the mucous and sub-mucus tissues covering the turbinated bones; on the other hand they differ from cavernous tissue.

These swellings frequently follow an acute attack of catarrh in a pre-existing chronic case; they may also follow an attack of catarrh when the chronic inflammation has not previously existed or has not been known by the patient to exist. In these latter cases the application of cocaine alone will usually reduce the enlargement though it may be necessary to scarify the more persistant tumors.

The condition appropriate for scarification can be differentiated from true hypertrophy by noting the sluggish recoil of the turgescent membrane when pressure upon it with a probe is suddenly discontinued.

When the knife is objected to I usually resort to the application of nitric acid saturated with muriate of cocaine for reducing these turgesences, but this means has not answered the purpose of the knife in my hands.

I have found scarifications especially serviceable in some Eustachian and middle ear inflammations that have been caused by hypertrophies or perhaps more properly, hyperplastic nasal catarrh from the fact that in many of these cases it is difficult to introduce the Eustachian catheter till the nasal calibre is enlarged; at least the catheter can then be used with less injury to the membrane, and sprays will prove effectual at the nasal end of the tube.

Patients have come to me to have their *red noses* treated when the cause has been other than the constant use of alcohol.

In these cases I have invariably found enlargements over the turbinated bones and they are usually soft and full of blood but not cavernous. All of these cases that have allowed systematic scarifications at intervals of a week for several months together with a spray treatment of pure terebene in cosmoline have been decidedly improved, not only in the enlargement of the calibre of the nose but in the lessening of the redness externally. In some cases the redness has entirely disappeared.

It may be said that the blood supply of the skin of the nose and its lining membrane is from different sources, nevertheless I can usually assure a patient that if I am able to overcome the determination of blood to the mucous tissues the external redness will decrease.

If it is desirable in any case after a scarification to stop the flow of blood, as I have occasionally found it to be, it may be easily done by pressing a large cotton probe saturated in a strong solution of cocaine firmly against the wound. In preparing the nose for a cut I much prefer the local application of the cocaine with a cotton probe, to the use of the spray, because of the unpleasant choking sensation that the latter sometimes produces when it reaches the throat. If no other purpose should be served than that of opening a way for a more thorough spraying of the deeper parts, scarification would be advisable when confined to the proper cases for its use.

